# Disparities by Race and Ethnicity in Cancer Survivor Stories Available on the Web

**DOI:** 10.2196/jmir.1163

**Published:** 2009-11-30

**Authors:** Katherine S Eddens, Matthew W Kreuter, Jennifer C Morgan, Kate E Beatty, Sina A Jasim, Lori Garibay, Donghua Tao, Trent D Buskirk, Keri A Jupka

**Affiliations:** ^5^Medical Center LibrarySaint Louis UniversitySt. LouisMOUSA; ^4^The University of Texas MD Anderson Cancer CenterHoustonTXUSA; ^3^Department of Community HealthSchool of Public HealthSaint Louis UniversitySt. LouisMOUSA; ^2^School of MedicineWashington University in St. LouisSt. LouisMOUSA; ^1^Health Communication Research Laboratory and Center for Cultural Cancer CommunicationGeorge Warren Brown School of Social WorkWashington University in St. LouisSt. LouisMOUSA

**Keywords:** Cancer, health disparities, survivorship, African American, Hispanic, online

## Abstract

**Background:**

The rapid growth of eHealth could have the unintended effect of deepening health disparities between population subgroups. Most concerns to date have focused on population differences in access to technology, but differences may also exist in the appropriateness of online health content for diverse populations.

**Objective:**

This paper reports findings from the first descriptive study of online cancer survivor stories by race and ethnicity of the survivor.

**Methods:**

Using the five highest-rated Internet search engines and a set of search terms that a layperson would use to find cancer survivor stories online, we identified 3738 distinct sites. Of these, 106 met study criteria and contained 7995 total stories, including 1670 with an accompanying photo or video image of the survivor. Characteristics of both websites and survivor stories were coded.

**Results:**

All racial minority groups combined accounted for 9.8% of online cancer survivor stories, despite making up at least 16.3% of prevalent cancer cases. Also notably underrepresented were stories from people of Hispanic ethnicity (4.1%), men (35.7%), survivors of colon cancer (3.5%), and older adults.

**Conclusions:**

Because racial/ethnic minority cancer survivors are underrepresented in survivor stories available online, it is unlikely that this eHealth resource in its current form will help eliminate the disproportionate burden of cancer experienced by these groups.

## Introduction

The benefits of eHealth information and services for patients and the public are well documented and numerous [1-8]. It is possible, however, that the growth of eHealth could have the unintended effect of deepening disparities in health status between population subgroups [9]. Most concerns in this regard have focused on population differences in access to technology, or the so-called “digital divide” [10]. More recently, research has focused on the availability of information over the Internet, or “infodemiology” [11,12]. But differences also exist in the *appropriateness* of health content available online for diverse population subgroups [13]. This issue has received much less attention in eHealth research and infodemiology and is the focus of the present study.

Specifically, the study explores the availability of online cancer survivor stories by race and ethnicity of the survivor. In the United States, cancer disproportionately affects African Americans, who are more likely than other groups to be diagnosed with cancer at a later stage of disease, who receive substandard cancer care once diagnosed, and who have lower 5-year survival rates and higher cancer death rates [14,15]. Similarly, Hispanic women have disproportionately high rates of cervical cancer, while Asian Americans and Pacific Islanders have higher rates of stomach and liver cancer [16]. Population-specific eHealth information and resources, such as online stories from cancer survivors representing these groups, might help address these disparities.

Survivors’ stories can model coping skills, provide perspective, and share valuable information and resources. An estimated 2.3 million persons with cancer are online [17], and use of information sharing and support sites, precisely where survivor stories are commonly available, is widespread [18-20]. Survivors’ direct experience and demonstrated success living with cancer makes them especially attractive and credible as messengers of cancer information [21], and studies have found psychological benefits for both the survivor and the recipient from sharing stories [22-24].

Race and ethnicity of a human information source are important factors in enhancing the effectiveness of communication, and specifically health communication, for members of minority populations. From a communication standpoint, the impact of information is generally enhanced when the recipient perceives the messenger as being similar to him- or herself. Similarity based on race, ethnicity, or other demographic characteristics can enhance receivers’ liking of an information source [25] and trust in the source [26], and it can lead to inferences of attitudinal similarity that in turn increase respect and perceived attractiveness of the source [27]. Advertising research shows that viewer responses to ads are more favorable when the models or actors in the ads are of the same race or ethnicity as the viewers [28,29]; this is especially true in minority groups like African Americans and Hispanics [30], and the effect is greatest among viewers who identify strongly with their racial/ethnic group [31,32].

These findings are reinforced by cancer control research showing that videos using race- and gender-concordant messengers can increase use of cervical cancer screening [33] and identification with a quit smoking role model [34] among minority women. In the specific case of cancer survivor stories, a recent study among African American women found that by far the strongest predictor of becoming engaged in a cancer survivor’s videotaped story and having positive reactions to the story was the extent to which participants saw themselves as similar to the survivor [21], including both attitudinal and demographic dimensions of similarity.

Finally, the importance of messenger characteristics is heightened when information is delivered via visual stimuli such as television, videos, and, increasingly, Web-based content. Studies show that compared to other media (eg, print, audio), video elicits more thoughts about and positive perceptions of a messenger [35,36], is better able to carry nonverbal messages [37], and is especially effective with messengers who are likable [38] or trustworthy [39,40]. In a meta-analysis of studies exploring source effects on persuasion, the size of such effects in visual media was exceeded only by face-to-face communication [41]. In short, the effects of race and ethnicity of a messenger will be greatest when these characteristics are apparent to audience members.

In summary, because survivor stories contain unique and valuable information, they may be especially useful to members of minority groups who suffer a disproportionate burden of cancer. Research from communication and persuasion suggests that audience members for such information are more likely to identify with and trust the survivor and act in accordance with the survivor’s story if they are of the same race or ethnicity. Thus, if stories from racially and ethnically diverse survivors were available online, we would generally expect that people exposed to these stories could find potential role models that were similar to them and who they trusted and liked, which would increase the probability that the information provided by the survivor was adopted and used.

This paper reports findings from the first descriptive study to document the availability of online cancer survivor stories by race and ethnicity of the survivor and to compare the results to expected population proportions and to cancer burden by race and ethnicity. As the number of online survivor stories grows and evidence of their benefits builds, it is important for assuring population health and achieving health equity that the diversity of survivors represented in these stories matches that of potential users.

## Methods

### Sampling

ComScore’s qSearch data were used to identify the five highest-ranked search engines at the time of data collection (October, 2007). ComScore is a global Internet information provider that maintains databases on real-time use of the Internet and consumer behavior in the Internet, and its qSearch tool measures all search activity on the Internet, including major search engines, private sites such as MySpace, vertical searches on sites such as Amazon or eBay, local searches for maps or directions, cross-channel searches such as searching the Web, maps and images for the same term, and more [42]. It is a comprehensive tool measuring the search universe, and it has been applied previously in scientific research [43]. The five top-ranked search engines were Google, Yahoo, Microsoft Sites (including MSN/Windows Live), Time Warner Networks (including AOL), and Ask Networks (including Ask.com) [42].

A subgroup of the study team generated a list of search terms that a layperson might use to search for online cancer survivor stories. This group included four family members of cancer patients, representatives of three different racial or ethnic groups, and an information science specialist with expertise in Web searching. The list took into consideration synonyms and word variations. A pilot search with each term was conducted, and the final six search phrases were selected because they yielded the largest number of relevant hits. The six search phrases used in the study were the following: “cancer survivor stor,” “cancer stor,” “cancer patient stor,” “cancer testimonial,” “sharing cancer stor,” and “cancer experience.” Boolean operator “OR” was used to perform the union of the six phrases. For cancers not identified by the word “cancer,” such as leukemia, lymphoma, and melanoma, we replaced the word “cancer” in the six search phrases with each of these, resulting in 18 total search phrases for online stories of survivors diagnosed with leukemia, lymphoma, and melanoma. We chose these three cancers because they may have been missed in our search and are also prevalent in the United States [44]. While searching “stor” in Google, AOL, and Ask.com can yield results with both “story” and “stories,” Yahoo and MSN/Windows Live do not accept the truncation search. Therefore, the words “story” and “stories” replaced “stor” in these search engines and resulted in doubling the total number of search phrases for these search engines. Summing all permutations of the original search terms, Google, AOL, and Ask.com had 24 distinct search phrases, while Yahoo and MSN/Windows Live had 48.

The search was performed from October 15 to October 30, 2007. Excluding sponsored links, the URLs of all websites were recorded until duplicates or irrelevant results dominated the search hits list, which resulted in 1420 websites identified from Google, 1055 from Yahoo, 1039 from MSN/Windows Live, 1055 from AOL, and 1053 from Ask.com. After removing exact duplicates, 3738 distinct websites were identified.

### Eligibility

To be included in the study, a website had to (1) contain cancer survivor stories in text, audio, or video form with an accompanying photo or video of the survivor; and (2) identify (or imply) presentation and/or sharing of stories as a purpose of the site. This latter criterion excluded personal blogs, news stories, and websites ending in a “PDF” extension, as these were usually reports. Websites hosted outside the United States were also excluded due to differences in cancer prevalence, racial/ethnic composition of the population, and use of different search engines. A total of 106 websites met these eligibility criteria.

### Coding

While many of the websites included stories without photos or video, we coded only those stories accompanied by a photo or video in which the survivor or storyteller was represented. Uncoded types of stories included those with text only, links to personal blogs, and stories entered on forums. On the 106 websites, there were 7995 stories total, of which 1670 (20.9%) had an accompanying photo or video image of the survivor.

Characteristics of websites and survivor stories were coded over a 2-month period from November 2007 to January 2008. Members of the research team who received formal training, rehearsal, and evaluative feedback completed all coding, adhering to specific operational definitions and coding instructions. The research team coded every cancer survivor story. In rare instances when a member of the research team had difficulty coding race or ethnicity, another trained coder was consulted and consensus reached.

### Measures

#### Website Characteristics

For each website, we counted the number of all human images and human images of minorities appearing on the website’s home page and (where applicable and different) on the home page for survivor stories. When minority images were present, they were coded using racial and ethnic categories from the 2000 US Census. Cancer site was coded as both a broad categorical type (a particular cancer, a set of related cancers, or general/all cancer) and by specific cancer (eg, lung cancer, breast cancer). Web address extension (.com, .edu, .org, etc) and the ability to post or share a story were also captured. The sponsoring organization of each website was recorded. Additionally, the total number of stories that were available on the website was recorded.

#### Survivor Characteristics

Survivor stories were coded for type of storyteller (cancer survivor, family member or friend of the survivor, or third person narrator such as the website editor or a journalist), survivor age at diagnosis, gender (when available in the story or discernible from an image), and survivor race and ethnicity (from story or images, using racial and ethnic categories from the 2000 US Census).

#### Form of Communication

Form of communication was recorded, including how the story content was presented (text or audio) and the type of image present (still image, video image, or link to photo). Both text and video stories could have accompanying audio tracks. Each image was coded for quality (professional photo/video vs a personal photo/video).

#### Cancer Type

Type of cancer, year of diagnosis, cancer stage, survival status, and years survived were also obtained, when available, from the content of the story. When information from story content or images was insufficient to make a definitive coding judgment for any measure, coders indicated so.

### Statistical Analyses

Descriptive statistics are provided to characterize survivor stories and websites. All stories and websites are included in the analysis.

## Results

### Website Characteristics

Characteristics of websites are summarized in Table 1. Of the 106 websites included in the final sample, 56 (52.8%) were hosted by nonprofit organizations (“.org”), 7 (6.6%) by educational institutions (“.edu”), and the remaining by for-profit companies, with Web extensions including “.com,” “.html,” and “.net.” Sixty-four of the 106 websites addressed cancer in general (60.4%), while the remainder focused on a specific type of cancer (n = 34, 32.1%) or a set of related cancers, such as leukemia and lymphoma or brain and other nervous system cancers (n = 8, 7.5%). The number of stories per website ranged from 1 to 232, with a mean of 16.5 (SD 32.2).

### Survivor Characteristics

Most stories were told by survivors (n = 1052, 63%), with the remainder told by a third person narrator (n = 390, 23.4%) or a family member or friend (n = 206, 12.3%). Most stories were told by women (n = 1073, 64.3%). When age was reported (n = 1008, 60.4% of stories), the mean age at diagnosis was 35 years. Characteristics of stories are summarized in Table 2.

### Form of Communication

Stories could be presented in more than one form. Nearly all stories were told through written text (n = 1643, 99.2%), with some told through audio (n = 337, 20.2%) and/or video (n = 264, 15.8%). Most stories were accompanied by a still photo (n = 1643, 98.4%), and half of these photos (n = 936, 56.0%) were professional grade.

### Cancer Type

One in four stories (n = 440, 26.3%) addressed breast cancer, followed by leukemia (n = 282, 16.9%), lymphoma (n = 165, 9.9%), prostate cancer (n = 142, 8.5%), and skin cancer, including melanoma, basal cell carcinoma, and squamous cell carcinoma (n = 69, 4.1%). Among stories that reported cancer stage at diagnosis (n = 318, 19.0%), later stages were most represented, with stage 4 diagnoses being most common (n = 88/318, 27.7%), followed by stage 3 (n = 85/318, 26.7%), stage 1 (n = 82/318, 25.8%), stage 2 (n = 57/318, 17.9%), and stage 0, (n = 6/318, 1.9%).

**Table 1:** Characteristics of websites containing cancer survivor stories (n = 106)

Characteristic	Percent^a^
**Type of cancer website**	
General cancer (n = 64)	60.4
Single cancer (n = 34)	32.1
Set of related cancers (n = 8)	7.5
**Searching and sharing capabilities**	
Users can post or share a story (n = 43)	40.6
Users can search library of stories with a search box (n = 11)	10.4
**Images**	
Images of racial/ethnic minorities on main landing page (n = 1501)	14.1
Images of racial/ethnic minorities on home page for stories (n = 1055)	14.6
**Web address extension**	
.org (n = 56)	52.8
.com (n = 37)	34.9
.edu (n = 7)	6.6
.htm(l) (n = 4)	3.7
.net (n = 2)	1.9
**Number of stories,** mean (SD)	16.5 (32.2)

^a^ Values are percentages unless otherwise noted.

**Table 2:** Characteristics of cancer survivor stories (n = 1670)

Characteristic	Percent^a^				
	Overall	White	Black	Asian^b^	Hispanic
**Survivor/storyteller**					
**Primary storyteller**					
Survivor (n = 1052)	63.0	62.8	68.0	61.3	61.8
Third person narrator (n = 390)	23.4	23.0	26.8	25.8	22.1
Family, friend, or caregiver (n = 206)	12.3	12.8	3.1	12.9	13.2
Could not be determined (n = 22)	1.3	1.3	2.1	0	2.9
**Gender of survivor**					
Female (n = 1073)	64.3	63.8	72.2	64.5	69.1
Male (n = 597)	35.7	36.2	27.8	35.5	30.9
**Other characteristics**					
Age in years at first cancer diagnosis (n = 1008), mean (SD)	35.0 (19.2)	35.1 (19.2)	38.0 (18.0)	29.0 (19.6)	35.4 (20.4)
Survivor living at time of story (n = 1670)	93.1	92.9	94.8	93.5	91.2
**Form of communication**					
**Story content**					
Text only (n = 1332)	79.8	80.4	82.5	64.5	79.4
Text and audio (n = 324)	19.4	18.8	17.5	35.5	20.6
Audio only (n = 13)	0.8	0.9	0	0	0
**Type of image present**					
Still image only (n = 1330)	79.6	79.9	81.4	75.8	88.2
Still image, video image, and link to photo (n = 172)	10.3	9.6	13.4	17.7	5.9
Still image and link to photo (n = 75)	4.5	4.7	1.0	1.6	2.9
Still image and video image (n = 66)	4.0	4.0	3.1	4.8	1.5
Video image only (n = 21)	1.3	1.3	1.0	0	0
Video image and link to photo (n = 5)	0.3	0.3	0	0	1.5
Link to photo only (n = 1)	0.1	0	0	0	0
**Image quality**					
Professional (n = 936)	56.0	55.9	57.7	53.2	45.6
Lay (n = 703)	42.1	42.2	41.2	46.8	48.5
Could not be determined (n = 31)	1.9	1.9	1.0	0	5.9
**Cancer type and stage**					
**Most common cancer types^c^**					
Breast (n = 440)	26.3	25.3	44.3	25.8	36.8
Leukemia (n = 282)	16.9	17.0	8.2	25.8	11.8
Lymphoma (n = 165)	9.9	9.7	8.2	17.7	8.8
Prostate (n = 142)	8.5	8.5	13.4	1.6	7.4
Skin (n = 69)	4.1	4.5	1.0	0	4.4
Brain and other nervous system (n = 66)	4.0	4.1	2.1	1.6	2.9
Ovary (n = 59)	3.5	3.7	1.0	1.6	1.5
Colon (n = 58)	3.5	3.4	3.1	6.5	2.9
Other (n = 363)	21.7	22.4	15.6	16.2	20.6
Unknown (n = 26)	1.6	1.4	3.1	3.2	2.9
**Stage at diagnosis (when reported; n = 318)**					
Stage 0 (n = 6)	1.9	1.7	14.3	0	0
Stage 1 (n = 82)	25.8	27.0	14.3	0	38.9
Stage 2 (n = 57)	17.9	17.7	14.3	22.2	11.1
Stage 3 (n = 85)	26.7	26.7	28.6	22.2	22.2
Stage 4 (n = 88)	27.7	27.0	28.6	55.6	27.8
**Race/ethnicity of survivors**					
**Race**					
White or White American (n = 1503)	90.0	-	-	-	-
Black or African American (n = 97)	5.8	-	-	-	-
Asian/Native Hawaiian/Other Pacific Islander (n = 62)	3.7	-	-	-	-
American Indian and Alaskan Native (n = 5)	0.3	-	-	-	-
Could not be determined (n = 3)	0.2	-	-	-	-
**Ethnicity^d^**					
Not Hispanic or Latino (n = 1524)	91.3	93.7	82.5	51.6	-
Hispanic or Latino (n = 68)	4.1	3.5	14.4	1.6	-
Could not be determined (n = 78)	4.7	2.8	3.1	46.8	-

^a^ Values are percentages unless otherwise noted.

^b^ Asian/Native Hawaiian/Other Pacific Islander.

^c^ *P* < .01.

^d^ *P* < .001.

### Differences by Race/Ethnicity

A large majority of stories in our sample (n = 1503, 90%) were told by whites. Among minority groups, blacks or African Americans were represented in 5.8% of stories (n = 97), Asians (including Native Hawaiian and Other Pacific Islanders) in 3.7% of stories (n = 62), and American Indian and Alaskan Natives in 0.3% of stories (n = 5). Race could not be determined in 0.2% of stories (n = 3). For ethnicity, most stories were from non-Hispanic or non-Latino survivors (n = 1524, 91.3%); 4.1% of survivors were identified as Hispanic or Latino (n = 68). In 4.7% of stories, the survivor’s ethnicity could not be determined (n = 78).

Most story characteristics did not differ across race or ethnicity. There was a significant difference in cancer type between races represented; however, with 33.3% of cells having counts less than five, the test statistic may not be valid.

## Discussion

Minority cancer survivors are underrepresented in survivor stories currently available online. African Americans make up 12.4% of the US population [45], account for 8.6% of prevalent cancer cases (limited duration prevalence, 0 to < 15 years since diagnosis [46]), and have higher overall cancer mortality rates than all other racial or ethnic groups [47], but, in this study, they accounted for just 5.8% of online survivor stories (n = 97). Similarly, persons of Hispanic origin account for 15.1% of the US population and 5.3% of prevalent cancer cases [46], yet make up just 4.1% of online survivor stories (n = 68). While reliable prevalence data are not available for all racial and ethnic minority groups, Asian/Pacific Islanders are properly represented in online survivor stories in this study, with 2.4% of prevalent cancer cases [46] and 3.7% of stories (n = 62).

A combination of differences in online access and patterns of eHealth usage across racial and ethnic groups likely explains at least part of this disparity. While socioeconomic status remains an important determinant of Internet access via personal computer [48], minorities are less likely than whites to have such access even in the lowest income groups [49-51]. Among those who have online access, studies suggest that minority group members are less likely to participate in online cancer support groups [52] or use the Internet for obtaining health information [53]. If exposure to and use of such stories are indeed less common among minority cancer survivors, we would generally expect these groups to have lower rates of story sharing as well, at least on websites where survivors could post their own stories. For example, while some of the websites in the study allowed users to post their personal stories, this would only happen among those who could and did access the site.

But on most websites in the study sample, the collection of available stories was set by the host and was not open to posting from users. On some websites, we found a large discrepancy between the level of racial and ethnic diversity represented on the home page and the comparative lack thereof in the actual collection of stories (overall, the two rates were comparable: 14.1% vs 13.7%, respectively). One interpretation of these cases is that website hosts recognize the value of offering stories from a diverse set of survivors (and thus give their site the outward appearance of diversity), but find it more difficult to identify minority survivors and/or collect their stories for sharing. To more consistently deliver on the promise and appearance of diversity suggested by websites’ home pages, hosts will likely need to take purposeful steps and consider different approaches to their story collection process. For example, access to racial and ethnic minority survivors might be increased by establishing partnerships with cancer care organizations serving these groups.

The disproportionate number of stories from young cancer survivors was unexpected and striking, even given the study’s methods. While cancer affects people of all ages, it is predominantly an older person’s disease. Yet the mean age of survivors who shared their stories was only 35, which is a full three decades younger than the median age at diagnosis for all cancers combined [47]. While a primary goal of this study was to determine whether race- and ethnicity-concordant survivor stories were available to minority cancer patients, this finding suggests that an even greater age gap may exist between those with cancer and those survivors whose stories are available online. Because sharing one’s story online with accompanying photos or video requires some degree of computer savvy (which studies have shown is currently inversely related to age [50]), this finding is not altogether surprising. It is possible, for example, that this study’s requirement of stories including a visual image of the survivor disproportionately excluded older survivors. It is also possible that those survivors whose stories did not disclose age at diagnosis (662/1670, or 39.6% in this sample) were older. Anecdotal information supports this latter explanation: we observed that in many stories from cancer survivors who were diagnosed at a young age, their age at diagnosis was highlighted as a kind of warning (eg, “I was only 28, I never thought this could happen to me”). Framing a story in this way would be less likely among older adults. While these two factors may account for some portion of the differences found, we think it is unlikely that they would entirely negate the finding, given the magnitude of the difference.

The finding that certain groups were underrepresented in survivor stories could also reflect the target audiences of the websites coded. Of the 106 websites, 39 were targeted at a specific survivor audience (eg, young adults or survivors of a specific cancer), and 42 were aimed at providing testimonials for a particular center, treatment, or product. Ten of the sites were targeted toward women, but only three were aimed at an audience of men. Perhaps most telling is that four sites were aimed specifically at younger adults, while none appear to be aimed at older adults, and none were specifically directed toward a minority group.

### Limitations

As this discussion has already identified, there are limitations to the study. Cancer survivor stories that did not include pictures of the survivor were not part of the sample. While this was necessary to achieve the study aim (ie, to identify race and ethnicity of survivors whose stories are available online), it’s possible that stories with and without pictures varied in other ways not intended in the study. If minority survivors were less likely than other survivors to provide a picture with their story, the study findings would underestimate the proportion of such stories. We have no indication whether survivor stories with and without pictures varied systematically by race or ethnicity. However, from a practical standpoint, unless story content explicitly mentioned the survivor’s race or ethnicity, this information would not be available to an online information seeker who might value it. Thus, while any real differences might be of interest for academic purposes, they would be largely irrelevant to those consuming the stories.

We also acknowledge that making judgments of a survivor’s race and ethnicity from online photos was sometimes challenging and, like any coding, subject to misclassification. In cases when multiple coders could not determine or agree upon a survivor’s race or ethnicity, it was classified as such. But because there were so few cases where race and ethnicity could not be determined (3/1670, 0.2% for race; 78/1670, 4.7% for ethnicity), it is improbable that misclassification bias alone would account for the pattern of findings in the study, even if every survivor whose race or ethnicity could not be classified was in fact a minority group member. Finally, it is possible, but we think highly unlikely, that stories from minority survivors exist in proportionally greater numbers under different search terms than those used in the study.

Thirty-two percent of the stories (534/1670) were hosted by four large not-for-profit organizations promoting patient advocacy and research. Each of these organizations has a public face that may draw more survivors from a variety of racial/ethnic backgrounds and age groups. Another 14.7% of stories (246/1670) were on websites of prominent cancer research and treatment centers. Some websites and organizations are doing a better job than others in recruiting minority cancer survivors to share their stories of survival. Organizations providing this service can learn from the websites that have collected libraries of stories from diverse populations. We also recognize that three of the four primary cancers represented in these stories (breast, leukemia, and lymphoma) do not occur disproportionately in minorities, and, therefore, it may not be surprising that we did not find a larger proportion of stories from minorities.

Besides increasing the proportion of survivor stories from minorities, older adults, and men, the study findings also suggest that websites providing cancer survivor stories might enhance their offering in at least three other ways. First, our research team learned that finding survivor stories online was often challenging and time consuming, requiring study team members to search through a lot of other cancer content to find stories. Stories were seldom available from a single location on a website, and the location of stories varied greatly from site to site.

Second, we observed that few sites (11/106, 10.4%) offered users a means of searching available cancer survivor stories, and less than half (43/106, 40.6%) allowed users to share or post a story.

Third, although we found stories from survivors of a wide range of cancers, there were clear gaps in the distribution of cancers represented. For example, only 58/1670 (3.5%) of stories were from colon cancer survivors, despite colon cancer being the third leading cause of cancer death in United States [54]. In addition, stories from lung and bronchus cancer survivors, the leading cause of cancer death in the United States for both men and women, represented only 1.6% of stories in this sample (n = 27). It may be that those with lung and bronchus cancer do not live very long and therefore don’t contribute to survivor stories on the Internet. We did find that when stage at diagnosis was reported, most of the survivors (173 of 318, 54.4%) were diagnosed with stage 3 or 4 cancers. Websites that select stories to post may choose stories from long-term survivors and from survivors who have overcome greater odds. Making improvements in these areas would enhance the accessibility and benefit of stories to users, as would developing technology tools that facilitate story sharing.

### Future Implications

The study also raises new questions and identifies promising avenues for future inquiry. An important next step may be determining the relative importance of technological (eg, online access, digital camera ownership, computer skills), psychological (eg, interest and willingness), and organizational (eg, website policies) factors in explaining the dearth of online survivor stories from minorities, older adults, and men. Intervention and audience research studies among cancer survivors might explore strategies to raise awareness of, interest in, and motivation to share online stories. Such work would be especially valuable when conducted among groups that were underrepresented in the online collections of stories in the current study (eg, racial and ethnic minorities, older adults, men, those with certain types of cancer). Usability research could test alternative Web designs and functionality to optimize ease and efficiency of use for sharing and accessing survivor stories.

We know from previous research that when characteristics of survivors match those of the reader or viewer, the information provided by the survivor will be more engaging, enhance information recall, and stimulate more thoughts about the story [21]. While these are important communication outcomes, they are several steps removed from the kinds of prevention, screening, or treatment adherence behaviors that would actually reduce cancer disparities. Still, matching the race/ethnicity of survivor stories to a viewer would seem to be an important and achievable first step toward these higher order outcomes.

Although there are currently no published studies reporting numbers of minority survivors seeking online survivor stories, there is considerable evidence that minority groups want and need better health information and often turn to the Internet to find it. The Internet is an important source of health information for African Americans and other minority groups, including patients diagnosed with cancer [55,56,58,59]. Yet cancer survivors as a group—including African Americans and Hispanics—are more likely than healthy adults to report wanting more information, having difficulty finding desired information, feeling frustrated during their search for information, and finding the information too hard to understand [57]. Because of differences in access to and use of the Internet in minority populations, changing the mix of stories alone won’t be enough to make an impact on cancer disparities. Changes in policy that would improve access to the Internet are also needed in addition to a better mix of survivor stories.

### Conclusions

This study provides the first descriptive summary of online cancer survivor stories and identifies some important gaps in currently available offerings. There is a risk that the benefits these stories can confer to users might be unequally distributed across the population due to a lack of stories from members of certain groups. The fact that several of these underrepresented groups also bear a disproportionate burden of cancer suggests that the collection of survivor stories available online today is unlikely to help eliminate disparities in cancer.
